# Supplementary material for: A Critical View of the Application of the APEX Software (Aqueous Photochemistry of Environmentally-Occurring Xenobiotics) to Predict Photoreaction Kinetics in Surface Freshwaters
Source: Molecules. 2019 Dec 18;25(1):9. doi: 10.3390/molecules25010009 (PMC7017383; doi:10.3390/molecules25010009)
Supplement: Supplementary file 1 [file molecules-25-00009-s001.zip › 3.2.4_gcc-4.4.0.zip!/3.2.4_gcc-4.4.0/3.2.4_gcc-4.4.0/doc/octave/html/interpreter/Three_002ddimensional-Function-Plotting.html]

Three-dimensional Function Plotting - Untitled


Next: Three-dimensional Geometric Shapes,
Up: Three-Dimensional Plotting


---

##### 15.1.2.1 Three-dimensional Function Plotting

— Function File: **ezplot3** (fx, fy, fz)  
— Function File: **ezplot3** (..., dom)  
— Function File: **ezplot3** (..., n)  
— Function File: **ezplot3** (h, ...)  
— Function File: h = **ezplot3** (...)  
> Plots in three-dimensions the curve defined parametrically.
> fx, fy, and fz are strings, inline functions
> or function handles with one arguments defining the function. By
> default the plot is over the domain `-2*pi <` x `< 2*pi`
> with 60 points.
>
> If dom is a two element vector, it represents the minimum and maximum
> value of t. n is a scalar defining the number of points to use.
>
> The optional return value h provides a list of handles to the
> the parts of the vector field (body, arrow and marker).
>
> ```
>           fx = @(t) cos (t);
>           fy = @(t) sin (t);
>           fz = @(t) t;
>           ezplot3 (fx, fy, fz, [0, 10*pi], 100);
> ```
>
>
>
>
> **See also:** plot3, ezplot, ezsurf, ezmesh.

— Function File: **ezmesh** (f)  
— Function File: **ezmesh** (fx, fy, fz)  
— Function File: **ezmesh** (..., dom)  
— Function File: **ezmesh** (..., n)  
— Function File: **ezmesh** (..., 'circ')  
— Function File: **ezmesh** (h, ...)  
— Function File: h = **ezmesh** (...)  
> Plots the mesh defined by a function. f is a string, inline
> function or function handle with two arguments defining the function. By
> default the plot is over the domain `-2*pi <` x `< 2*pi` and
> `-2*pi <` y `< 2*pi` with 60 points in each dimension.
>
> If dom is a two element vector, it represents the minimum and maximum
> value of both x and y. If dom is a four element vector,
> then the minimum and maximum value of x and y are specify
> separately.
>
> n is a scalar defining the number of points to use in each dimension.
>
> If three functions are passed, then plot the parametrically defined
> function `[`fx `(`s`,` t`),` fy `(`s`,` t`),` fz `(`s`,` t`)]`.
>
> If the argument 'circ' is given, then the function is plotted over a disk
> centered on the middle of the domain dom.
>
> The optional return value h provides a list of handles to the
> the parts of the vector field (body, arrow and marker).
>
> ```
>           f = @(x,y) sqrt(abs(x .* y)) ./ (1 + x.^2 + y.^2);
>           ezmesh (f, [-3, 3]);
> ```
>
> An example of a parametrically defined function is
>
> ```
>           fx = @(s,t) cos (s) .* cos(t);
>           fy = @(s,t) sin (s) .* cos(t);
>           fz = @(s,t) sin(t);
>           ezmesh (fx, fy, fz, [-pi, pi, -pi/2, pi/2], 20);
> ```
>
>
>
>
> **See also:** ezplot, ezsurf, ezsurfc, ezmeshc.

— Function File: **ezmeshc** (f)  
— Function File: **ezmeshc** (fx, fy, fz)  
— Function File: **ezmeshc** (..., dom)  
— Function File: **ezmeshc** (..., n)  
— Function File: **ezmeshc** (..., 'circ')  
— Function File: **ezmeshc** (h, ...)  
— Function File: h = **ezmeshc** (...)  
> Plots the mesh and contour lines defined by a function. f is a string,
> inline function or function handle with two arguments defining the function.
> By default the plot is over the domain `-2*pi <` x `< 2*pi` and
> `-2*pi <` y `< 2*pi` with 60 points in each dimension.
>
> If dom is a two element vector, it represents the minimum and maximum
> value of both x and y. If dom is a four element vector,
> then the minimum and maximum value of x and y are specify
> separately.
>
> n is a scalar defining the number of points to use in each dimension.
>
> If three functions are passed, then plot the parametrically defined
> function `[`fx `(`s`,` t`),` fy `(`s`,` t`),` fz `(`s`,` t`)]`.
>
> If the argument 'circ' is given, then the function is plotted over a disk
> centered on the middle of the domain dom.
>
> The optional return value h provides a list of handles to the
> the parts of the vector field (body, arrow and marker).
>
> ```
>           f = @(x,y) sqrt(abs(x .* y)) ./ (1 + x.^2 + y.^2);
>           ezmeshc (f, [-3, 3]);
> ```
>
>
>
>
> **See also:** ezplot, ezsurfc, ezsurf, ezmesh.

— Function File: **ezsurf** (f)  
— Function File: **ezsurf** (fx, fy, fz)  
— Function File: **ezsurf** (..., dom)  
— Function File: **ezsurf** (..., n)  
— Function File: **ezsurf** (..., 'circ')  
— Function File: **ezsurf** (h, ...)  
— Function File: h = **ezsurf** (...)  
> Plots the surface defined by a function. f is a string, inline
> function or function handle with two arguments defining the function. By
> default the plot is over the domain `-2*pi <` x `< 2*pi` and
> `-2*pi <` y `< 2*pi` with 60 points in each dimension.
>
> If dom is a two element vector, it represents the minimum and maximum
> value of both x and y. If dom is a four element vector,
> then the minimum and maximum value of x and y are specify
> separately.
>
> n is a scalar defining the number of points to use in each dimension.
>
> If three functions are passed, then plot the parametrically defined
> function `[`fx `(`s`,` t`),` fy `(`s`,` t`),` fz `(`s`,` t`)]`.
>
> If the argument 'circ' is given, then the function is plotted over a disk
> centered on the middle of the domain dom.
>
> The optional return value h provides a list of handles to the
> the parts of the vector field (body, arrow and marker).
>
> ```
>           f = @(x,y) sqrt(abs(x .* y)) ./ (1 + x.^2 + y.^2);
>           ezsurf (f, [-3, 3]);
> ```
>
> An example of a parametrically defined function is
>
> ```
>           fx = @(s,t) cos (s) .* cos(t);
>           fy = @(s,t) sin (s) .* cos(t);
>           fz = @(s,t) sin(t);
>           ezsurf (fx, fy, fz, [-pi, pi, -pi/2, pi/2], 20);
> ```
>
>
>
>
> **See also:** ezplot, ezmesh, ezsurfc, ezmeshc.

— Function File: **ezsurfc** (f)  
— Function File: **ezsurfc** (fx, fy, fz)  
— Function File: **ezsurfc** (..., dom)  
— Function File: **ezsurfc** (..., n)  
— Function File: **ezsurfc** (..., 'circ')  
— Function File: **ezsurfc** (h, ...)  
— Function File: h = **ezsurfc** (...)  
> Plots the surface and contour lines defined by a function. f is a
> string, inline function or function handle with two arguments defining the
> function. By default the plot is over the domain `-2*pi <` x `<
> 2*pi` and `-2*pi <` y `< 2*pi` with 60 points in each dimension.
>
> If dom is a two element vector, it represents the minimum and maximum
> value of both x and y. If dom is a four element vector,
> then the minimum and maximum value of x and y are specify
> separately.
>
> n is a scalar defining the number of points to use in each dimension.
>
> If three functions are passed, then plot the parametrically defined
> function `[`fx `(`s`,` t`),` fy `(`s`,` t`),` fz `(`s`,` t`)]`.
>
> If the argument 'circ' is given, then the function is plotted over a disk
> centered on the middle of the domain dom.
>
> The optional return value h provides a list of handles to the
> the parts of the vector field (body, arrow and marker).
>
> ```
>           f = @(x,y) sqrt(abs(x .* y)) ./ (1 + x.^2 + y.^2);
>           ezsurfc (f, [-3, 3]);
> ```
>
>
>
>
> **See also:** ezplot, ezmeshc, ezsurf, ezmesh.
